# Astrocyte Store-Released Calcium Modulates Visual Cortex Synapse Development and Circuit Function

**DOI:** 10.21203/rs.3.rs-7890067/v1

**Published:** 2025-11-17

**Authors:** Gillian Imrie, Jordan Mar, Madison B. Gray, Isabella Farhy-Tselnicker

**Affiliations:** 1 Department of Biology, Texas A&M University, College Station, TX 77843, USA; 2 Texas A&M Institute for Neuroscience (TAMIN), Texas A&M University, College Station, TX 77843, USA; 3 Center for Biological Clocks Research, Texas A&M University, College Station, TX 77843, USA

**Keywords:** Astrocyte, calcium, IP3R2, visual circuit development, synapse development

## Abstract

Astrocytes, a major class of glial cells, are critical regulators of neuronal synapse development and function. Deficits in astrocyte-synapse interactions are implicated in various neurological disorders, yet the precise mechanisms by which astrocytes guide synapse formation and maturation during development remain poorly understood. A key astrocytic mechanism for integrating both extrinsic (e.g., neuronal) and intrinsic signals is the IP3-mediated release of intracellular calcium (Ca^2+^) from endoplasmic reticulum (ER) stores, modulating a wide range of downstream effects. Though defects in this signaling pathway have been linked to adult brain dysfunction, its role in shaping synaptic development, a period when astrocyte-neuron communication is established, is largely unknown. Here, we investigate the role of IP3-mediated Ca^2+^ signaling in astrocyte-dependent regulation of synapse development in the mouse visual system. Using a combination of histological, molecular, and circuit-level approaches, we find that loss of the IP3 receptor (IP3R type 2; IP3R2) leads to significant deficits in the maturation of glutamatergic but not GABAergic synapses. These synaptic disruptions are accompanied by attenuated visually evoked neuronal activation and impaired behavioral responses to visual threat stimuli. We further show that astrocytic morphological complexity is diminished in the absence of IP3R2, suggesting that store-released Ca^2+^ is required for both the structural and functional maturation of astrocyte-neuron interactions. Our findings establish a critical role for astrocytic IP3R2-mediated Ca^2+^ signaling in shaping excitatory circuit development and the emergence of visually driven behaviors.

## INTRODUCTION

Synapses, the fundamental units of electrochemical communication in the brain, are tightly regulated by astrocyte activity throughout all life stages^1–3^. During early postnatal development, when excitatory and inhibitory neuronal circuits are actively forming and refining, astrocytic regulatory functions influence the maturation, strength, and specificity of synaptic connections^2,4,5^. Dysregulation of synapse formation is linked to numerous brain pathologies, including depression and mood disorders^6^, autism spectrum disorders^7^, and epilepsy^8^; however, the precise mechanisms by which astrocytes regulate synapse formation and maturation are not yet fully understood.

In the mouse visual cortex (VC), synapse development and maturation follows a well-defined postnatal trajectory starting at around postnatal day (P) 7, peaking at P14 concurrently with eye opening, and stabilizing near P28^2^. Astrocyte development is temporally aligned with these synaptogenic stages^2,9,10^, during which astrocytes engage in robust bidirectional communication with neurons and undergo major changes in gene expression and structural morphology which promote the appropriate spatiotemporal recruitment of synaptic components in a neuronal activity-dependent manner^4,11^. This allows astrocytes to dynamically signal in a rapidly changing environment and to integrate neuronal activity in ways appropriate to developmental stage and context^4,11,12^. Though the importance of these developmental interactions has been described in previous work^2–5,13^, the exact signaling pathways activated in astrocytes to modulate them remain unresolved.

A central mechanism underlying astrocytic responses to intrinsic and extrinsic cues occurs via fluctuations in intracellular calcium (Ca^2+^). Astrocytic Ca^2+^ signals arise from diverse sources, exhibit complex spatial and temporal dynamics, and have been associated with multiple regulatory functions from the cellular to behavioral levels^14–17^, including energy metabolism, neuronal circuit synchronization, network scaling, and encoding of sensory stimulus details like salience and context^18–23^. A major pathway by which astrocytes integrate neuronal signals involves Ca^2+^ release from endoplasmic reticulum (ER) stores^24^. Neurotransmitters and neuromodulators bind to astrocytic G-protein coupled receptors (GPCRs), activating phospholipase C to generate inositol 1,4,5-trisphosphate (IP3). IP3 binds to its receptors on the ER, which in cortical astrocytes are predominantly IP3 receptors type 2 (IP3R2), triggering Ca^2+^ release into the cytosol modulating various downstream effects^12,25–32^. The physiological role of IP3R2-mediated Ca^2+^ signaling remains somewhat controversial, as while some studies link its disruption to functional deficits like cortical dysregulation and autism-like behaviors^4,17,22,26,32,33^, others report no effects^34,35^. This conflicting evidence likely stems from the use of variant knockout strategies, developmental stages, and diverse brain regions across different studies. Importantly, most of the work investigating IP3R2-mediated signaling has been focused on adults, while little is known about the involvement of this signaling pathway in modulating synapses during development, a time when astrocyte-neuronal communication is established. Thus, thorough investigation into the brain region-, circuit-, and developmental stage-specific functions of this signaling pathway is needed.

Here, we determine the role of IP3R2-mediated store-released Ca^2+^ signaling in the astrocyte-dependent regulation of synapse development within the mouse visual circuit. Using a combination of histological, molecular, and circuit level approaches across defined postnatal timepoints, we demonstrate that loss of astrocytic IP3R2 leads to deficits in the maturation of glutamatergic but not GABAergic synapses in the primary visual cortex (VC). These synaptic disruptions are accompanied by attenuated visually evoked neuronal activation across multiple visual circuit-relevant brain regions, and impaired behavioral responses to visual threat stimuli. We further show that astrocyte morphological complexity in the VC is diminished in the absence of IP3R2 at P16, a timepoint critical for astrocytic synapse regulation and processing of visual information following eye opening, suggesting that store-released Ca^2+^ is required for both the structural and functional maturation of astrocyte-neuron interactions. Our findings establish a critical role for astrocytic IP3R2-mediated Ca^2+^ signaling in shaping the development of excitatory circuits and visually driven behaviors.

## RESULTS

**Figure 1.**
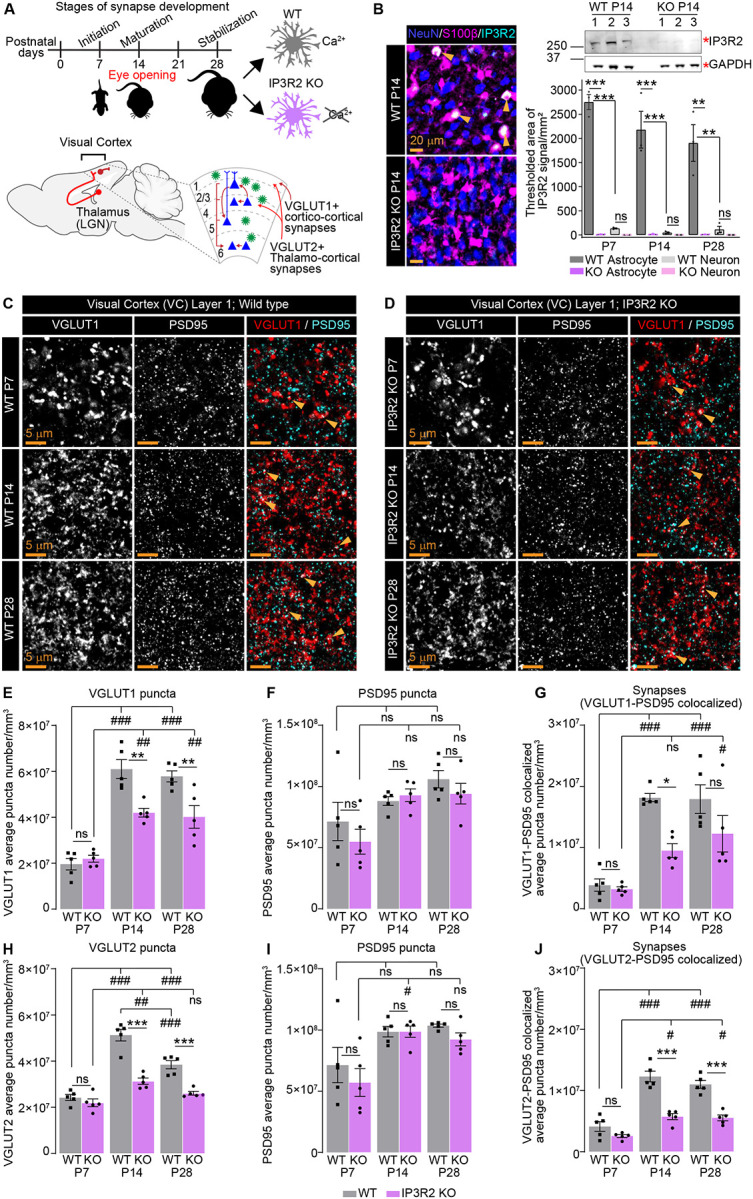
Glutamatergic synapse development is perturbed in IP3R2 KO mice VC. See also Figure S1, Table S1. **A.** Schematic of experiment: Brain tissue is collected from WT and KO mice at P7, P14 and P28 corresponding to stages of synapse development; IHC to quantify synapses as indicated is performed in Layer 1 of the VC for VGLUT1-containing cortico-cortical synapses, and VGLUT2-containing thalamo-cortical synapses. **B.** Validation of IP3R2 KO by IHC and WB (top right panel). Example images of IP3R2 (cyan), astrocyte marker S100β (magenta) and neuronal marker Neun (blue) in the VC at P14 as labeled. Graph on the right is quantification of colocalized signal with each cell marker. IP3R2 signal is highly colocalized with astrocytes and not with neurons and is downregulated in KO VC. Top panel is WB showing IP3R2 band (~250KDa) and GAPDH (loading control, ~36 KDa) in WT and KO at P14 as labeled. Numbers indicate samples from individual animals. Uncropped blots are shown in Fig. S1D-F. **C-G.** VGLUT1-containing synapses are reduced in IP3R2 KO VC at P14 and P28 but not P7. Example images of the presynaptic VGLUT1, postsynaptic PSD95 and merged (synapses) in each age and genotype as labeled (**C-D**), and quantification of individual synaptic proteins and synapse number per mm^3^ represented as colocalization between VGLUT1 and PSD95 for both genotypes (**E-F**) are shown. Single channel grayscale images on the left, merged images on the right. The number of VGLUT1 puncta and VGLUT1-containing synapses is reduced in IP3R2 KO (**E, G**), while PSD95 numbers are unaltered (**F**). **H-J.** VGLUT2-containing synapses are reduced in IP3R2 KO VC at P14 and P28 but not P7. Quantification of individual synaptic proteins and synapse number per mm^3^ represented as colocalization between VGLUT2 and PSD95 for both genotypes are shown (see also Fig. S1G-H). The number of VGLUT2 puncta and VGLUT2-containing synapses is reduced in KO (**H, J**), while PSD95 numbers are unaltered (**I**). Graphs show mean ± s.e.m. Individual mouse data points are shown as circles and squares. Number of mice/group (N) N=3 in B; N=5 in E-J. Scale bar = 20 μm in B; 5 μm in C-D. Arrowheads mark representative IP3R2 signal colocalized with astrocyte marker S100β in B, and colocalized puncta in C-D. ^#^,*P≤0.05; ^##^,**P<0.01; ^###^,***P<0.001 by one-way ANOVA with post-hoc Tukey’s test. ^#^ - indicates comparisons between age groups within each genotype, * - indicates comparisons between WT and KO in E-J; and comparison between WT astrocytic and neuronal groups in B; ns denotes non-significant results (P>0.05).

Astrocytic intracellular Ca^2+^ fluctuations are fundamental to their regulatory roles at the neuronal synapse. While these Ca^2+^signals are characterized by a diversity of forms and origins^36,37^, store-released Ca^2+^ mediated by the astrocytic IP3R2 couples synaptic neurotransmitter release to changes in synapse regulating genetic programs in astrocytes^4,38^. We previously identified that mice lacking IP3R2, which is essential for astrocytic store-released Ca^2+^ signaling, have dysregulated expression of synapse modulating genes including Glypican 4 and Chordin like 1, and decreased numbers of vesicular glutamate transporters at P14^4^. To investigate whether synapses in IP3R2 knockout (KO) mice are broadly dysregulated across development, we profiled the expression of pre- and post-synaptic proteins in layer 1 of the visual cortex (VC) of wild-type (WT) and IP3R2 KO mice at P7, P14, and P28 which correspond to cortical synaptogenic onset, peak, and stabilization respectively^2^ (Fig. 1A). We first validated the astrocytic expression of IP3R2 and its deletion in the KO mice at the mRNA and protein levels using real-time quantitative PCR (qPCR), immunohistochemistry (IHC), and western blot (WB) across these developmental stages (P7, P14, P28) (Fig. 1B, Fig. S1A-F). IP3R2 mRNA (gene name *Itpr2*) was undetectable in IP3R2 KO mice VC (shown as mean ± s.e.m. [ΔCt relative to GAPDH]: WT 5.09 ±0.16; KO 0.00 ±0.00; Fig. S1C). For IHC, brain sections were co-labeled with astrocyte specific marker S100β and neuronal specific marker Neun to determine cell-type specificity of IP3R2 expression. Our results show colocalization of IP3R2 signal with S100β but not with Neun, confirming the astrocytic source of this receptor within the developing VC gray matter. Furthermore, we observed a strong reduction of IP3R2 protein levels in KO tissue by both IHC and WB, confirming this model’s validity for our experiments (IHC data shown as mean ± s.e.m. [thresholded area mm^2^]: IP3R2-S100β colocalized [P7] WT 2752 ±156.70, KO 11.05 ±5.51; [P14] WT 2180 ±380.4, KO 13.48 ±7.38; [P28] WT 1905 ±379.9, KO 10.71 ±3.19; IP3R2-Neun colocalized [P7] WT 135.4 ±10.31, KO 1.25 ±1.20; [P14] WT 47.31 ±15.91, KO 3.66 ±1.08; [P28] WT 112.60 ±68.56, KO 0.35 ±0.14; Fig. 1B, Fig. S1A-B; WB data is shown as mean ± s.e.m normalized to GAPDH: [P7] WT 0.13 ±0.007, KO 0.002 ±0.001; [P14] WT 0.15 ±0.01, KO 0.02 ±0.009; [P28] WT 0.06 ±0.003, KO: 0.01 ±0.0005; Fig. 1B, Fig. S1D-F).

### Vesicular glutamate transporters and synapse numbers are developmentally reduced in the visual cortex of IP3R2 knockout mice

To determine how glutamatergic synapse development is impacted by IP3R2 KO, we used IHC to quantify synapses of two glutamatergic circuits within the VC, the presynaptic vesicular glutamate transporter 1 (VGLUT1) specific cortico-cortical circuit and vesicular glutamate transporter 2 (VGLUT2) specific thalamo-cortical circuit together with the excitatory postsynaptic scaffolding protein, postsynaptic density protein 95 (PSD95). We quantified both the numbers of pre- and postsynaptic puncta and their colocalizations to calculate synapse number across developmental stages^4^ (Fig. 1C–J, Fig. S1G-H). We observed a significant decrease in VGLUT1 (~30%) and VGLUT2 (~40%) at P14 (in accordance with our previous work)^4^ which persisted to P28 in VC sections from IP3R2 KO mice (~30% for both proteins) (data shown as mean ± s.e.m. [puncta number per mm^3^]: VGLUT1 [P14] WT 6.10E7 ±4.08E6, KO 4.20E7 ±1.89E6, [P28] WT 5.78E7 ±2.40E6, KO 4.02E7 ±4.92E6; VGLUT2 [P14] WT 4.92E7 ±2.43E6, KO 2.99E7 ±1.32E6, [P28] WT 3.68E7 ±1.72E6, KO 2.49E7 ±7.76E5; Fig. 1E, H), with no change at P7 (VGLUT1 WT 1.96E7 ±2.53E6, KO 2.20E7 ±1.49E6; VGLUT2 WT 2.31E7 ±1.25E6, KO 2.09E7 ±1.63E6; Fig. 1E, H). Similarly, synapse numbers were decreased for both VGLUT1 and VGLUT2-containing projections at P14 (~41% and 35% respectively) and P28 (~32% and 50% respectively) (VGLUT1-PSD95 colocalized [P14] WT 1.82E7 ±6.72E5, KO 9.51E6 ±1.13E6; [P28] WT 1.79E7 ±2.34E6, KO 1.23E7 ±2.98E6; VGLUT2-PSD95 colocalized [P14] WT 1.23E7 ±8.69E5, KO 5.73E6 ±4.93E5; [P28] WT 1.10E7 ±6.39E5, KO 5.53E6 ±4.77E5; Fig.1G, J), with no difference at P7 (VGLUT1-PSD95 colocalized WT 3.87E6 ±1.01E6, KO 3.22E6 ±3.71E5; VGLUT2-PSD95 colocalized WT 4.14E6 ±7.92E5, KO 2.55E6 ±2.41E5; Fig. 1G, J). The observed synaptic deficits were driven by the reduction in presynaptic proteins, as levels of PSD95 were unchanged between WT and IP3R2 KO mice at all ages tested ([P7] WT (from VGLUT1 data) 7.14E7 ±1.57E7; (from VGLUT2 data) 7.14E7 ±1.44E7, KO (from VGLUT1 data) 5.49E7 ±1.01E7; (from VGLUT2 data) 5.71E7 ±1.13E7; [P14] WT (from VGLUT1 data) 8.81E7 ±3.64E6; (from VGLUT2 data) 9.86E7 ±4.29E6, KO (from VGLUT1 data) 9.28E7 ±5.20E6; (from VGLUT2 data) 9.87E7 ±4.59E6; [P28] WT (from VGLUT1 data) 1.06E8 ±7.09E6; (from VGLUT2 data) 1.04E8 ±1.25E6, KO (from VGLUT1 data) 9.41E7 ±8.42E6; (from VGLUT2 data) 9.24E7 ±5.30E6; Fig. 1F, I).

To further analyze how VGLUTs are disrupted in IP3R2 KO mice we quantified the sizes of individual puncta (represented as total volumes from Imaris 3D rendering, see Methods) and computed the cumulative frequency of VGLUT puncta volumes for WT and IP3R2 KO mice at each age (Fig. S2A-F). We observed significantly reduced VGLUT1 and VGLUT2 volumes at P14 (by ~40%, and ~35% respectively) and P28 (by ~14% and ~20%) in IP3R2 KO mice compared to WT (VGLUT1 [P14] WT 1.12 ±0.009, KO 0.66 ±0.006, [P28] WT 1.0 ±0.01, KO 0.86 ±0.01; VGLUT2 [P14] WT 0.79 ±0.008, KO 0.51 ±0.007; [P28] WT 0.80 ±0.009, KO 0.64 ±0.008; Fig. S2B-C, E-F), with no difference at P7 (data shown as mean ± s.e.m. [volume μm^3^]: VGLUT1 WT 0.89 ±0.01, KO 0.84 ±0.01; VGLUT2 WT 0.83 ±0.01, KO: 0.78 ±0.01; Fig. S2A, D). These data suggest that IP3R2 mediated Ca^2+^ signaling is necessary for the developmental maintenance, but not initiation, of glutamatergic synapses in the VC.

### Axonal density and cell number are unaffected by IP3R2 knockout

**Figure 2.**
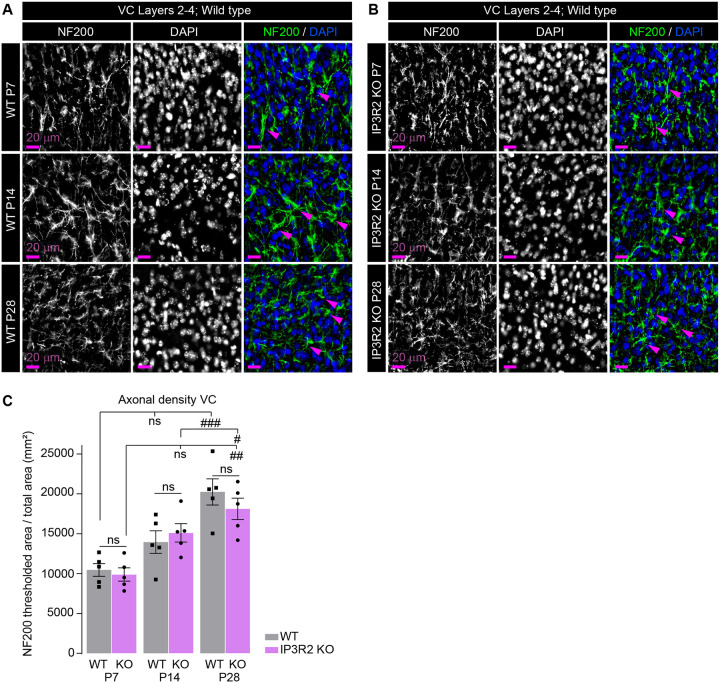
Axonal density is unperturbed in IP3R2 KO mice VC. See also Figure S2, Table S1. **A-B.** Example VC images of the axonal marker NF200 (green) and nuclear marker DAPI (blue) in each age and genotype as labeled. Single channel grayscale images on the left, merged images on the right. **C.** Quantification of NF200 signal represented as thresholded area of signal for each age genotype as labeled. Axonal density is similarly increased in both WT and KO from P7 to P28. No difference between WT and KO is observed at any age. Graph shows mean ± s.e.m. Individual mouse data points are shown as circles and squares. Number of mice/ group (N) N=5. Scale bar = 20 μm. Arrowheads mark representative axons. ^#^P≤0.05; ^##^P<0.01; ^###^P<0.001 by one-way ANOVA with post-hoc Tukey’s test comparing NF200 signal between age groups within each genotype. ns denotes non-significant (P>0.05) results.

The observed decrease in VGLUT1 and VGLUT2 immunoreactivity could be indicative of reduced cortical and thalamic innervation to the VC or overall reduction in cell numbers due to decreased cell survival or proliferation during development. To test this, we immunolabeled VC sections from WT and IP3R2 KO mice at P7, P14, and P28 for the axonal intermediate filament protein neurofilament 200 (NF200) to quantify axonal density^39^ (Fig. 2A–B; Fig. S2H). Axonal density increased steadily across developmental stages supporting previous studies^40,41^, with no changes observed between WT and IP3R2 KO mice at any of the timepoints analyzed (data shown as mean ± s.e.m. [thresholded area mm^2^]: [P7] WT 10460 ±803, KO 9883 ±836.3, [P14] WT 13953 ±1414, KO 15108 ±1163, [P28] WT 20239 ±1646, KO 18117 ±1339; Fig. 2C). To analyze cell density in the VC, we quantified cells marked by nuclear label DAPI. Cell density decreased across development in accordance with our previous work^4^, and did not differ between WT and KO groups (data shown as mean ± s.e.m. [cell number per mm^2^]: [P7] WT 4504 ±253.7, KO 4186 ±120.5, [P14] WT 2274 ±99.58, KO 2262 ±101.2, [P28] WT 2275 ±221.1, KO 2221 ±230.1; Fig. S2G). To examine how axonal and cell density are affected by IP3R2 KO in regions outside the VC, we quantified NF200 and DAPI density in the Schaffer collaterals of the hippocampus and similarly, observed no differences between WT and KO groups (data shown as mean ± s.e.m. NF200 [thresholded area mm^2^]: WT 13424 ±887.4, KO 13366 ±1067; DAPI [cell number per mm^2^]: WT 2376 ±187.4, KO 2329 ±59.76; Fig. S2H-J). These results suggest that the developmental synaptic protein deficiencies observed in IP3R2 KO mice VC are synapse-specific, and not due to an overall decrease in cell numbers or axonal innervation.

### Inhibitory synapse development in the visual cortex is unaltered by IP3R2 knockout

**Figure 3.**
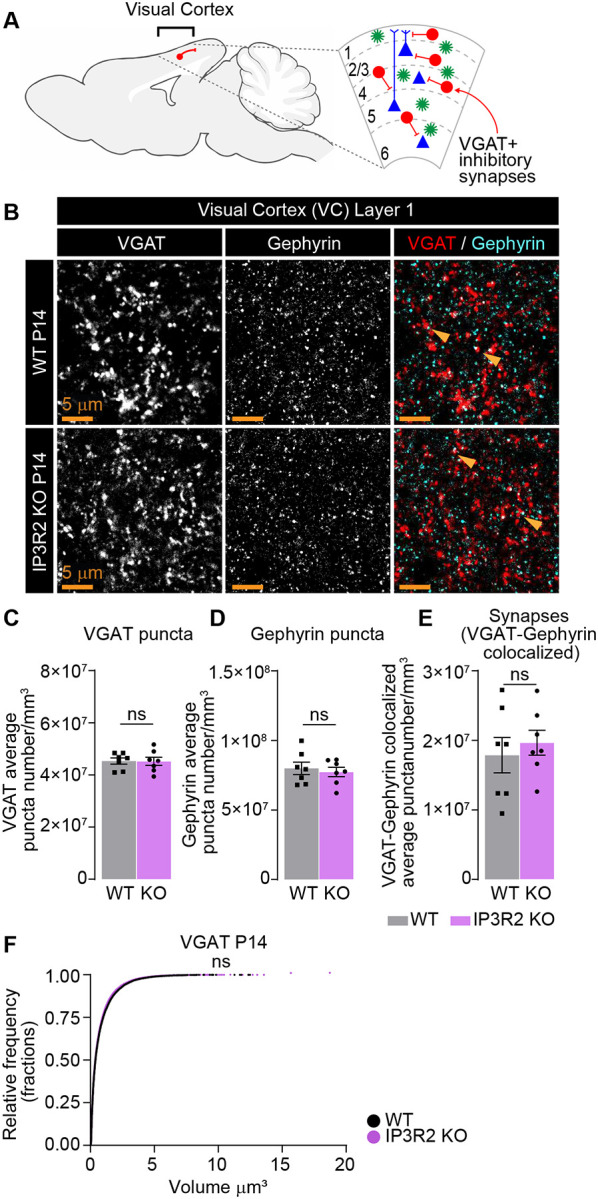
GABAergic synapses are unperturbed in IP3R2 KO mice VC at P14. See also Table S1. **A.** Diagram depicting inhibitory neurons within the VC analyzed. **B-F.** GABAergic synapse numbers are not altered in IP3R2 KO VC at P14. Example images of the presynaptic VGAT, postsynaptic Gephyrin and merged (synapses) in each genotype as labeled (**B**) and quantification of individual synaptic proteins and synapse number per mm^3^ represented as colocalization between VGAT and Gephyrin for both genotypes (**C-E**) are shown. Single channel grayscale images on the left, merged images on the right. No difference is observed in any of the parameters compared. **F.** Cumulative distributions of volumes from 3D rendered images for VGAT per genotype as labeled. Bar graphs show mean ± s.e.m. Individual mouse data points are shown as circles and squares. Number of mice/ group (N) N=5. Scale bar = 5 μm. Arrowheads mark representative colocalized puncta. ns denotes non-significant results (P>0.05) by t-test in C-E, and Kolmogorov-Smirnov test in F, comparing WT and KO groups.

The mouse VC is composed of ~80% glutamatergic and 20% GABAergic neurons^42^. To test whether the observed synaptic deficits are specific to excitatory circuits we used IHC to label pre-synaptic vesicular GABA transporters (VGAT) and the inhibitory post-synaptic scaffolding protein Gephyrin in WT and IP3R2 KO mice at P14 (Fig. 3A, B), the timepoint at which we observed the greatest deficits in glutamatergic synapses (Fig. 1C–J; Fig. S1G-H). There was no difference between WT and KO groups in any of the parameters quantified including VGAT puncta number (shown as mean ± s.e.m. [puncta number per mm^3^]: WT 4.53E7 ±1.18E6, KO 4.52E7 ±1.58E6; Fig. 3C), Gephyrin puncta number (WT 8.00E7 ±4.40E6, KO 7.73E7 ±3.36E6; Fig. 3D), synapses - VGAT-Gephyrin colocalized (WT 1.79E7 ±2.54E6, KO 1.20E7 ±1.79E6; Fig. 3E), or VGAT volumes (shown as mean ± s.e.m. [volume μm^3^]: WT 0.80 ±0.007, KO 0.80 ±0.006; Fig. 3F). These results demonstrate that developmental synaptic disruptions in IP3R2 KO mice are specific to glutamatergic terminals in the VC.

### Visually evoked upregulation of neuronal immediate early gene c-FOS is blunted in IP3R2 knockout visual circuits

**Figure 4.**
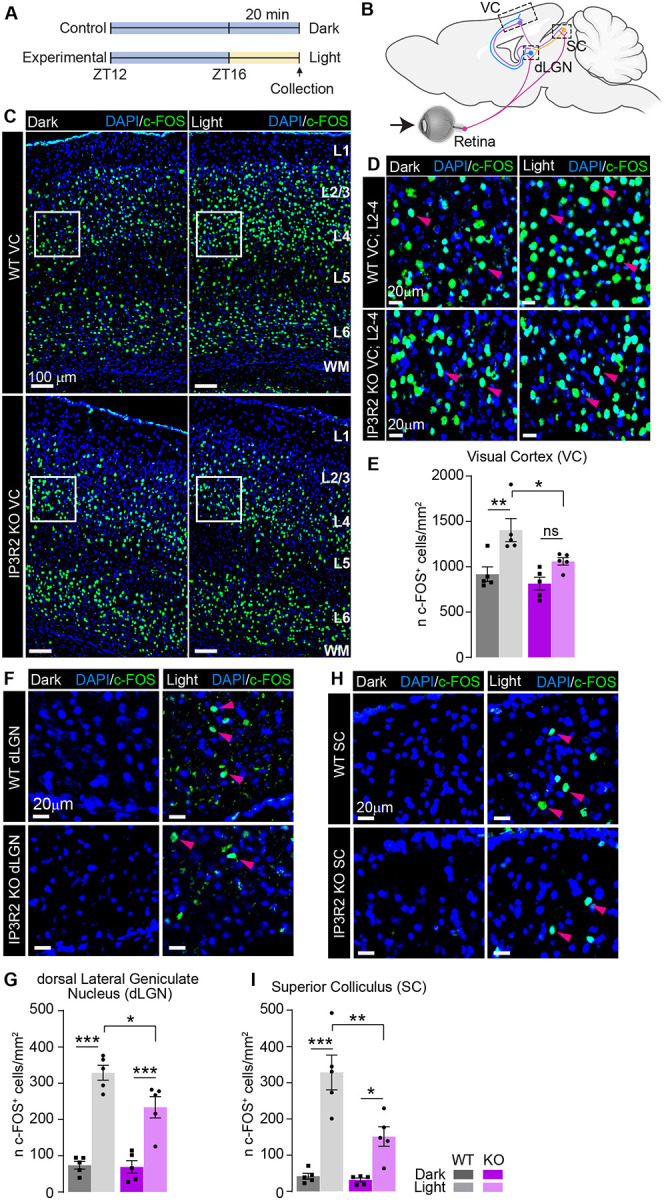
Light-induced c-FOS expression is blunted in IP3R2 KO mice visual circuit. See also Figure S4, Table S1. **A.** Schematic of experimental paradigm. Following 4 hours of dark exposure (ZT12 marks lights off), mice were exposed to 20-minute light pulse, tissue collected immediately after for IHC analysis of c-FOS expression. **B.** Diagram of the visual circuit components including the retina (which receives the light input) and downstream connections to the superior colliculus (SC) and dorsal Lateral Geniculate Nucleus (dLGN), which input to the Visual cortex (VC). **C-E.** Light evoked c-FOS levels are diminished in IP3R2 KO mice VC. **C.** Example images of the cortex including all cortical layers as labeled on the right showing c-FOS (green) and nuclear marker DAPI (blue) in each genotype as labeled in the dark or light exposed groups. **D.** Zoomed in images from box in C as labeled showing Layers 2–4. **E.** Quantification of C-B represented as numbers of c-FOS labeled cells per area (c-FOS cell density). Light exposure induces a strong increase in c-FOS density in the WT, but not IP3R2 KO VC. **F-I.** Same as C-E but for the dorsal lateral geniculate nucleus of the thalamus (dLGN; **F-G**) and the superior colliculus (SC; **H-I**). In both regions, light exposure produced a strong increase in c-FOS positive cell density in the WT, and to a lesser extent in the KO. Graphs show mean ± s.e.m. Individual mouse data points are shown as circles and squares. Number of mice/ group (N) N=5. Scale bar in B = 100 μm; in D, F, H = 20 μm. Arrowheads mark representative c-FOS positive cells. *P≤0.05, **P<0.01, ***P<0.001 by one-way ANOVA with post-hoc Šidák’s test. ns denotes non-significant results (P>0.05).

Reduction in excitatory synaptic proteins in the VC could contribute to deficits in visually evoked neuronal activity, which has been shown to undergo regulation by astrocyte Ca^2+^ fluctuations^4,36^. To test whether disruption of store released Ca^2+^ in astrocytes affects the activation of neurons in the VC, we examined activity-dependent expression of the protein product of the immediate early gene *c-fos* (protein name c-FOS) in response to a brief light pulse stimulus following prolonged darkness (4hours; Fig. 4 A) in 16-day old mice (to ensure mice had at least 24 hours of processing visual information following eye opening at ~P14). Fluorescent immunolabeling of c-FOS protein in the VC revealed that in WT mice, light stimulation induced a robust increase in the number of c-FOS positive cells (~50%) compared to control group kept in the dark (shown as mean ± s.e.m. [cell number per mm^2^]: WT dark 917.4 ±80.38, light 1404 ±127.9; Fig. 4C–E). Because distinct cortical layers receive and integrate different streams of visual input and output (Fig. 1A, 4B, and^4^), we also analyzed c-FOS induction separately across layers (Fig. S4A-E) and observed significantly increased expression in L2/3 (~50%) which integrates local input response from other layers and L4 (~40%) which receives visual input from the thalamus (WT [L2/3] dark 892.7 ±63.37, light 1649 ±107, [L4] dark 1512 ±143.6, light 2090 ±170.9; Fig. S4B-C). In the IP3R2 KO VC this effect was blunted, showing weaker light-induced c-FOS expression (~20–30%; statistically significant only in L2/3), and significantly lower light-induced expression than WT in both total VC and Layer 2/3 (KO [VC] dark 814.2 ±69.86, light 1059 ±41.08; Fig. 4D, E; [L2/3] dark 764.1 ±112.1, light 1135 ±98.36, [L4] dark1424 ±77.12, light 1676 ±79.47; Fig. S4B-C). Notably, no difference in c-FOS expression was observed between WT and IP3R2 KO in the control (dark) condition, suggesting that basal level of neuronal activation is not perturbed by IP3R2 removal. The observed effects were not due to changes in cell number as evident from similar DAPI-positive signal across the genotypes and light/dark conditions (data shown as mean ± s.e.m. [cell number per mm^2^]: [L1] WT dark 1360 ±42.84, light 1430 ±83.73; KO dark 1420 ±70.53, light 1281 ±63.49; [L2/3] WT dark 2879 ±113.5, light 3069 ±212.2; KO dark 2911 ±86.31, light 2790 ±201.8; [L4] WT dark 3377 ±324, light 3801 ±283.6; KO dark 3162 ±190.8, light 2916 ±252.3; [L5] WT dark: 2523 ±202.8, light 2201 ±182.4; KO dark 2678 ±220.4, light 2414 ±348.7 [L6] WT dark 3724 ±281, light 3617 ±225.4; KO dark 3444 ±343.9, light 3207 ±279.1; Fig. S4I-N)

To test whether the observed attenuated activity in the VC reflects upstream changes in subcortical visual processing, we examined c-FOS expression in the dorsal lateral geniculate nucleus (dLGN) (Fig. 4F–G) and superior colliculus (SC) (Fig. 4H–I) which receive direct retinal input and relay visual information to the VC^43–46^ (Fig. 4B). WT mice exhibited dramatic increases in c-FOS positive cell numbers in both the dLGN (~340%) and SC (~680%) upon light stimulation (WT [dLGN] dark 73.99 ±10.67, light 328.9 ±20.64, [SC] dark 41.93 ±8.107, light 328.2 ±48.14; Fig. 4G, I). However, this increase was blunted in IP3R2 KO group showing ~320% increase in the dLGN and ~380% in the SC, significantly lower than in WT mice in both regions (KO [dLGN] dark 69.13 ±17.36, light 233.8 ±29.52 [SC] dark 31.80 ±5.31, light 151.5 ±26.94; Fig. 4G, I). Like in the VC, these c-FOS changes were not due to differences in cell numbers between WT and IP3R2 KO mice as evident from similar DAPI signal (WT [dLGN] dark 3074 ±61.99, light 3091 ±197.4; [SC] dark 3427 ±196.8, light 3463 ±196.1; KO [dLGN] dark 2487 ±159.8, light 2871 ±285.5; [SC] dark 3240 ±134.6, light 3400 ±146.6; Fig. S4Q-R). To determine whether the effects of the light pulse were vision-specific, we quantified the number of c-FOS positive cells in the CA1 and dentate gyrus (DG) regions of the hippocampus (Fig. S4F-H) and observed no changes in c-FOS protein induction, or DAPI-positive cell numbers between the genotypes or light/dark conditions, as expected (c-FOS signal WT [CA1] dark 1532 ±233.9, light 1992 ±365.5; [DG] dark 309.6 ±32.1, light 369.4 ±37.41; KO [CA1] dark 1324 ±254, light 1642 ±145; [DG] dark 295.0 ±44.34, light 288.1 ±40.27; Fig. S4G-H; DAPI signal WT [CA1] dark 5812 ±439.8, light 5194 ±473.5; [DG] dark 4416 ±200.4, light 4938 ±312.3; KO [CA1] dark 4683 ±527.3, light 4418 ±510.3; [DG] dark 4416 ±152.5, light 4241 ±375.8; Fig. S4O-P). These results indicate that IP3R2-mediated astrocytic Ca^2+^ signaling plays an important role in facilitating visually evoked, but not basal, neuronal activity across brain regions comprising the visual circuit.

### Visually evoked behavioral responses are diminished in IP3R2 knockout mice

**Figure 5.**
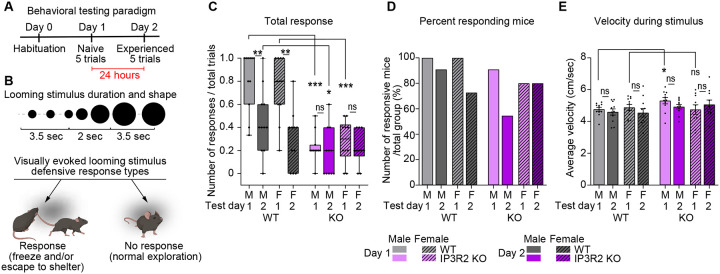
Visually evoked defensive behavior is disrupted in IP3R2 KO mice. See also Figure S5, Table S1. **A.** Diagram of the behavioral experiment including habituation and 2 testing days performed 24 hours apart. Each testing day includes 5 trials of the looming stimulus. Mice are naïve to the stimulus on Day 1 and retested on Day 2 (experienced). **B.** Schematic of the looming stimulus shape and duration (top) and the types of defensive responses analyzed (bottom). **C.** Average defensive responses (of any type, Total response) across genotypes, sexes, and experimental days. WT mice of both sexes exhibit robust response to the looming stimulus on Day1 and reduced response when retested (Day 2). KO mice responses are strongly downregulated compared to WT, showing no difference between testing days. **D.** Number of responders represented as percentage of mice responding to at least one trial per day out of total group number. All groups had similar percentage of mice responding to the stimulus. **E.** Velocity averaged across the entire 9 seconds of the stimulus, shows similar kinematic parameters for both sexes and genotypes. In C, graph shows box with range, line is median. In D, graph shows percentage of responding mice out of total group. In E, graph shows mean ± s.e.m. Individual mouse data points are shown as circles and squares. Number of mice (N) N=8–10/ sex/ genotype. *P≤0.05; **P<0.01; ***P<0.001 by Mann-Whitney test. ns denotes non-significant results (P>0.05).

Since we observed circuit level deficits in visually evoked neuronal activation, we next asked whether these findings translate to alterations in the development of behaviors which rely on these circuits for appropriate execution. Visually evoked defensive responses depend on an animal’s ability to see the threatening stimulus and integrate that information to coordinate an appropriate behavioral output^47,48^. We hypothesized that if IP3R2 KO mice experienced reduced or delayed activation of visual circuit neurons in response to salient stimuli at P16 (Fig. 4) and showed significantly reduced excitatory synapse number at P28 (Fig. 1G, J) when synaptogenesis is stabilized, they may exhibit disrupted behavioral responses to visual stimuli. To test this hypothesis, we subjected 4-week-old WT and IP3R2 KO mice (P30) to a visual threat paradigm. During this task, mice are presented with a visual shadow stimulus that mimics a looming predator^49–51^. Responses were quantified across two testing days, over which animals were considered naïve or adapted to the stimulus (experienced), respectively (Fig. 5A–B). Responses were scored (1 – response, 0 – no response) and further categorized into four different response types: “Freeze” – mice exhibit immobility; “Escape” – mice move towards and enter the shelter; “Freeze+Escape” – immobility followed by movement towards and entering the shelter; “No response” – mice continue exploratory behavior such as movement or sniffing (Fig. 5B; see Methods).

WT mice of both sexes (males [M], females [F]) responded robustly to the looming stimulus (regardless of response type, termed Total response) on Day 1 (D1, naïve) and significantly reduced their responses on Day 2 (D2, experienced) (shown as median and [range]: WT M D1 1.0 [0.3–1.0], D2 0.4 [0–1.0]; F D1 0.8 [0.2–1.0], D2 0.4 [0–0.8]; Fig. 5C), demonstrating high sensitivity to the visually triggered looming stimulus and rapid adaption upon re-exposure. In contrast, naïve IP3R2 KO mice had strongly abrogated responses relative to WT, and lack of behavioral adaptation between test days (KO M D1 0.2 [0–0.5], F D1 0.3 [0–0.5]; M D2 0.2 [0–0.6], F D2 0.2 [0–0.4]; Fig. 5C). There was no difference between M and F responses for either genotype. We then separately analyzed the four different response types in both genotypes and sex groups (Fig. S5A-C). The “Escape” response type is highest (average response; Fig. S5B) and most frequent (percentage of total responses per day; Fig. S5D) in both WT and KO mice on D1 compared to “Freeze” or “Freeze+Escape”, and is significantly higher in WT mice compared to IP3R2 KO (“Escape” average response shown as median and [range]: WT M D1 0.4 [0–1.0], D2 0.4 [0–0.8]; F D1 0.4 [0–1.0], D2 0.2 [0–0.5]; KO M D1 0.2 [0–0.5], D2 0 [0–0.6]; F D1 0.2 [0–0.5], D2 0.2 [0–0.4]; Fig. S5B). Conversely, “Freeze” responses did not differ between genotypes (average response: WT M D1: 0 [0–0.2], D2: 0 [0–0.4]; F D1: 0 [0–0.2], D2: 0 [0–0.2]; KO M D1: 0 [0–0], D2: 0 [0–0.2]; F D1: 0 [0–0.2], D2: 0 [0–0.2]; Fig. S5A), while “Freeze+Escape” responses were undetected in IP3R2 KO mice on any testing day (average response: WT M D1: 0.2 [0.0–0.6], D2: 0 [0–0.2]; F D1: 0.2 [0–0.6], D2: 0 [0–0.6]; KO M D1: 0 [0–0], D2: 0 [0–0]; F D1: 0 [0–0], D1: 0 [0–0]; Fig. S5C; Percentage of different responses out of total response number per day: “Escape”: WT M D1 53.84%, D2 34.54%; F D1 44.44%, D2 18.86%; KO M D1 22.64%, D2 15.68%; F D1 20.83%, D2 18%; “Freeze”: WT M D1 7.69%, D2 5.45%; F D1 5.55%, D2 3.77%; KO M D1 0%, D2 3.92%; F D1 6.25%, D2 4%; “Freeze+Escape”: WT M D1 21.15%, D2 5.45%; F D1 24.07%, D2 7.54%; KO M D1 0%, D2 0%; F D1 0%, D2 0%; Fig. S5D)

To determine responsiveness at the group level, we quantified the percent responding mice (represented as mice who respond to at least one trial per day) out of total group number and observed no difference between WT and IP3R2 KO groups of either sex across test days for Total response (WT M D1 100%, D2 91%; F D1 100% D2 73%; KO M D1 91%, D2 55%, F D1 80%, D2 80%; Fig. 5D) “Freeze” response (WT M D1 36%, D2 18%, F D1 27%, D2 18%; KO M D1 0%, D2 18%, F D1 30%, D2 20%; Fig. S5E) or “Escape” responses (WT M D1 91%, D2 82%, F D1 91%, D2 64%; KO M D1 91%, D2 36%, F D1 70%, D2 60%; Fig. S5F). Conversely, percent responders by “Freeze+Escape” is significantly higher in WT group, with none of the KO mice exhibiting this response type, in accordance with our average response data (WT M D1 73%, D2 27%, F D1 64%, D2 64%; KO M D1 0%, D2 0%, F D1 0% D2 0%; Fig. S5G). These results suggest that the reduced responses in KO mice are not stemming from decreased responses from select individuals, but rather, a group-wide reduced responsiveness. Importantly, average velocity during the stimulus was similar overall across the experimental groups, suggesting that the observed responses were not due to differences in ambulatory abilities (shown as mean ± s.e.m velocity [cm/sec]: WT – M D1: 4.77 ±0.12, D2: 4.60 ±0.19, F D1: 4.86 ±0.19, D2: 4.55 ±0.27; KO – M D1: 5.31 ±0.20, D2: 4.93 ±0.14, F D1: 4.76 ±0.30, D2: 5.07 ±0.29; Fig. 5E). These findings demonstrate that IP3R2 KO mice exhibit an impairment in visually evoked defensive behaviors, despite normal mobility, supporting a role for astrocytic store-released Ca^2+^ signaling in shaping behavior through experience-dependent circuit refinement.

### Astrocyte morphology is abnormal in IP3R2 knockout mice visual cortex

**Figure 6.**
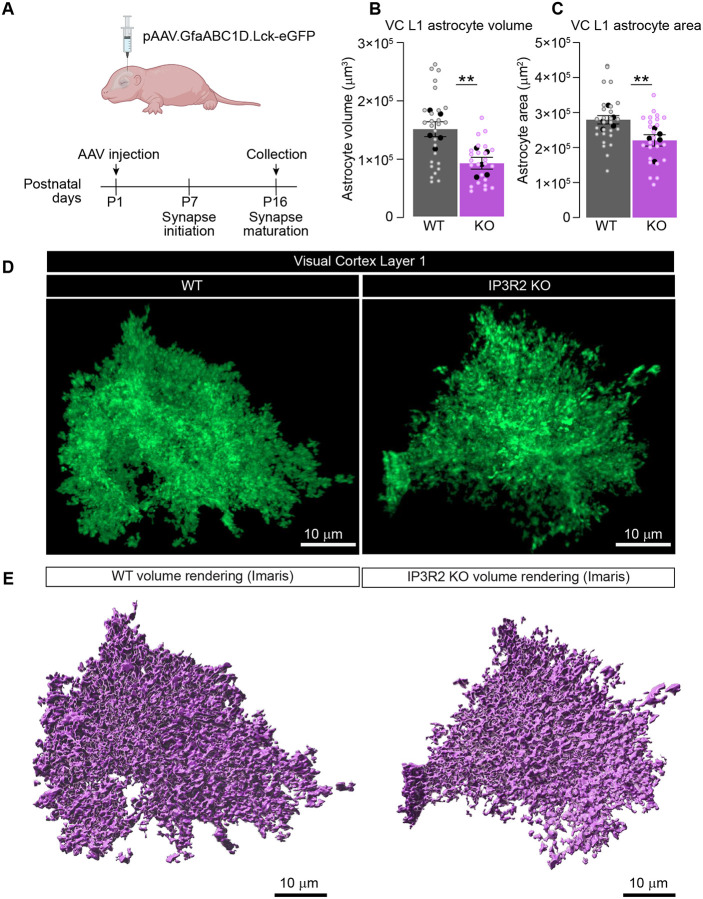
Reduced morphology in IP3R2 KO astrocytes. See also Figure S6, Table S1. **A.** Schematic of the strategy to express membrane tethered fluorescent reporter in astrocytes. P1 mouse pups injected intracerebroventricularly (icv) with AAV to express membrane tethered eGFP (Lck-eGFP) under astrocyte specific promoter GfaABC1D. Tissue is collected 2 weeks following injection, sectioned, and imaged using super resolution confocal microscope (Zeiss Airyscan; see Methods). **B-E.** Astrocytic volume and area are reduced in IP3R2 KO mice compared to WT. Example images of Lck-eGFP (green) expressing astrocytes (**D**) and 3D rendering of volume using Imaris (**E**) for each genotype is shown as labeled. **B-C.** Quantification shows reduced total volume and area of IP3R2 KO astrocytes in L1 VC. Graphs show mean ± s.e.m. Black circles are average of signal in each mouse; colored open circles are data for each astrocyte. Number of mice/ group (N) N=5, number of astrocytes = 25–27. Scale bar = 10 μm. **P<0.01 by t-test.

An important process in development is the establishment of astrocytic morphological complexity, which is essential for effective synaptic ensheathment and support^1,52^. Therefore, it is plausible that the observed neuronal deficits in IP3R2 KO arise from astrocytes’ inability to fully and effectively interact with the developing synapses. Indeed, our previous work identified multiple differentially expressed genes in P14 IP3R2 KO mice VC known in the literature to be related to cellular metabolism and growth^4^ (Fig. S6A). We selected several genes for validation using qPCR in VC tissue from WT and IP3R2 KO mice and confirmed significant expression changes in a number of the candidates, further supporting our hypothesis (shown as mean ± s.e.m [ΔCt relative to GAPDH, normalized to WT]: *Glud1* 1.67 ±0.23; *Grin2b* 1.63 ±0.23; *Kcnk2* 1.78 ±0.26; Mapk10 1.59 ±0.15; Fig. S6B). To test for morphological disruptions in IP3R2 KO mice, we delivered an Adeno Associated Viral (AAV) vector via intracerebroventricular (icv) injection to sparsely express the membrane tethered fluorescent reporter (Lck-eGFP) under control of the minimal astrocytic promoter GfaABC_1_D^53^ at P1–2 and quantified astrocyte morphological development two weeks later at P16 (Fig. 6A). The membrane tethered reporter is necessary to fully capture astrocyte morphological complexity, while sparse labeling ensures the ability to analyze individual astrocytes. Lck-eGFP labeling was highly specific (100% of Lck-eGFP expressing cells in VC colocalized with S100β astrocytic marker) and sparse (~30% of S100β positive (^+^) cells expressed Lck-eGFP) validating our approach for these experiments (Fig. S6C-D). Cortical sections were imaged using super resolution confocal microscopy and resulting 3D volumetric renderings were constructed from the Lck-eGFP signal using Imaris (Fig. 6D–E; see Methods) and analyzed for size and geometric qualities. We observed a significant reduction in total astrocyte volume (~40%) in IP3R2 KO astrocytes in VC Layer 1 compared to WT (data shown as mean ± s.e.m [μm^3^]: WT 14980 ±1270, KO 9148 ±1011; Fig.6B) and in area (~20%) (shown as mean ± s.e.m [μm^2^]: WT 28004 ±1187, KO 22093 ±1624; Fig. 6C). There were no significant differences in oblate ellipticity (WT 0.51 ±0.02, KO 0.54 ±0.03; Fig. S6E), prolate ellipticity (WT 0.30 ±0.04, KO 0.28 ±0.04; Fig. S6F), or sphericity (WT 0.11 ±0.007, KO 0.10 ±0.005; Fig. S6G) between WT and IP3R2 KO astrocytes. These findings demonstrate that IP3R2 KO astrocytes have morphological deficits compared to WT at P16, a critical developmental timepoint for astrocytic regulation of synapse development. While overall cell size was significantly reduced, the general geometry of IP3R2 KO astrocytes remained consistent with WT astrocytes, suggesting that store-released astrocytic Ca^2+^ signaling modulates astrocyte outgrowth and elaboration while fundamental cytoskeletal and membrane patterning mechanisms are likely preserved.

## DISCUSSION

This study reveals that astrocytic IP3R2-mediated Ca^2+^ signaling is essential for the maturation and refinement of visual system synapses, circuits, and behavior. Specifically, we show:

- IP3R2 KO mice exhibit reduced numbers and volumes of presynaptic VGLUTs and VGLUT-containing synapses at P14 and P28, but not P7. These reductions are specific to glutamatergic presynaptic terminals, with no changes in the number of postsynaptic densities, axonal innervation to the visual cortex, total cell number, or inhibitory GABAergic synapses.
- Visually evoked neuronal c-FOS activation in response to a light pulse is reduced in IP3R2 KO mice across visual circuit brain regions, but baseline levels of neuronal c-FOS activity remain unaffected.
- Defensive behavioral responses to a visually triggered looming stimulus are diminished in IP3R2 KO mice.
- Astrocyte volume and area are decreased in the VC of IP3R2 KO mice at P16 during the period of peak synaptogenesis, concurrent with differential expression of cell-growth related genes.

### Astrocytic ER store-released Ca^2+^ supports synaptic development

The appropriate spatio-temporal expression of synaptic proteins is critical for proper synapse maturation in the developing brain. In IP3R2 KO mice, we observed a developmentally specific reduction of presynaptic VGLUTs (Fig. 1, Fig. S1, Fig. S2), expanding on our previous findings demonstrating a dysregulation in the expression of post synaptic AMPA receptor subunits GLUA1 and GLUA2 in IP3R2 KO VC at P14^4^. Accompanied by our results that IP3R2 protein levels were stable across development (Fig. 1B), this suggests that IP3R2 signaling becomes increasingly important as synapses mature. While initial stages of synaptogenesis in the VC may be driven by neuron autonomous programs or astrocytic Ca^2+^-independent mechanisms, later timepoints, such as the period following eye opening, likely require astrocytic input mediated by store-released Ca^2+^ signaling to stabilize functional connections. This aligns with previous work showing that astrocytic gene expression becomes increasingly specialized during specific stages of synapse development, and that Ca^2+^ dependent transcriptional regulation is a major mechanism by which astrocytes influence circuit assembly and responses to neuronal activity^4,12,25,32^.

Notably, synaptic deficits in IP3R2 KO mice were limited to glutamatergic circuits, with inhibitory synapses remaining intact (Fig. 3). This synapse type specific effect has important implications for cortical excitatory/inhibitory (E/I) balance. Disruptions in E/I ratio are a hallmark of multiple neurological disorders^54–57^, and previous studies have linked astrocyte Ca^2+^ signaling to their pathogenesis. For example, it was shown that IP3R2 KO mice exhibit disrupted resting-state functional connectivity in medial prefrontal cortex (mPFC)-centered networks, mirroring patterns observed in humans with major depressive disorder^58^, which is characterized by disrupted glutamate and GABA signaling^6^. Early life disruptions in glutamatergic synapses caused by dysregulated astrocytic store-released Ca^2+^ signaling could precede the development of these disorders in the mature brain. Research focusing on identifying the downstream Ca^2+^ dependent pathways in astrocytes that selectively influence excitatory synapse maturation and maintain E/I balance during critical developmental periods will be essential to deepening our understanding of these processes.

### IP3R2-mediated Ca^2+^ signaling contributes to the development of visual circuit function and behavioral output

At the circuit level, we found that evoked (but not basal) neuronal activation was impaired in IP3R2 KO mice. Expression of the immediate early gene c-FOS was significantly reduced in response to visual light stimulation across both cortical and subcortical regions of the visual circuit including the VC, dLGN, and SC (Fig. 4, Fig. S4). These findings are consistent with previous reports demonstrating that astrocytic Ca^2+^ transients contribute to stimulus dependent neuronal activation and cortical state transitions, particularly in sensory cortices where astrocytic Ca^2+^ activity has been shown to gate sensory throughput and regulate network gain^38,59^. Specifically, IP3R2 KO was shown to elevate sensory evoked gamma activity, an effect that reflects reduced astrocyte-mediated modulation of cortical excitability and aligns with our observation of impaired neuronal activation following visual stimulation. Further, the suppression of visually evoked c-FOS expression in IP3R2 KO mice is consistent with work showing that astrocyte mediated modulation of excitatory neurotransmission is critical for experience-dependent plasticity in early postnatal visual circuits^60^.

At the behavioral level, these circuit impairments translated into abrogated responses to a visual looming threat (Fig. 5, Fig. S5). These deficits may in part be caused by disruptions in evoked activity in both the visual cortex and the superior colliculus (Fig. 4), which is critical for the development and execution of defensive responses in mice and humans^61,62^. Future studies leveraging circuit level manipulations will be necessary to determine whether these effects arise from alterations in top-down processing from the visual cortex, or from deficits intrinsic to the superior colliculus. These findings further align with reports of disrupted social behaviors in adult IP3R2 KO mice, including delayed dominance behaviors, impaired social interaction, and autism spectrum disorder-like phenotypes in both germline and astrocyte specific IP3R2 KO models, underscoring the prominent astrocytic contributions to these behaviors^22,32^. Furthermore, it was shown that the *onset latency* of social dominance behavior, but not the overall behavior itself, was disrupted in IP3R2 KO mice^32^. Both defensive and social interactions require cortical integration of salient sensory cues to generate appropriate behavioral outputs. In accordance, we observed that defensive response types were differentially modulated in IP3R2 KO mice (Fig. S5). The selective loss of “Freeze+Escape” response type suggests a deficit in coupling sensory input with context-dependent behavioral output, or an impairment in the ability of IP3R2 KO mice to transition from one behavioral state to the next, which may contribute to social impairments identified in other studies^22,32^. Together, these observations suggest a potential common mechanism in which disrupted astrocytic Ca^2+^ signaling compromises the integration of sensory information with appropriate behavioral outcomes.

Such selective disruptions may also help reconcile why some studies have reported no discernable changes in synaptic activity or behavior in IP3R2 KO mice^34,35^. Behavioral outputs are highly modular in nature, with specific astrocytic events contributing to different components of a behavior, such as network tuning required for a behavior to begin or end. Thus, while astrocytic store-released Ca^2+^ is probably not responsible for every aspect of the behavioral outputs that it is involved in, it likely plays important roles in shaping their specific components and temporal dynamics, an idea consistent with recent reports implying that astrocytic Ca^2+^ is important to behavioral state transitions^63–66^. Moreover, some astrocytic Ca^2+^ linked behaviors occur entirely independent of IP3R2 mediated signaling^26^, emphasizing a need for studies that quantify how different types of astrocytic Ca^2+^ signals integrate to produce functional outputs^17^. Future specified studies will be necessary to determine how astrocytic Ca^2+^ signaling interfaces with neuromodulatory systems and whether restoring evoked responses in IP3R2 deficient circuits can rescue behavioral deficits.

### Store-Released Ca^2+^ Regulates Astrocyte Morphological Development

A major unresolved question involves the potential mechanisms by which astrocytes may induce their regulatory effects on synaptic maturation, and the role that store-released Ca^2+^ plays in this function. We approached this question by assessing gene expression changes in IP3R2 KO mice and identified broad dysregulation in cell growth and metabolism related genes. We further quantified astrocytic morphogenesis at P16, observing reduced volume and area of VC KO astrocytes (Fig. 6, Fig. S6), suggesting a possible mechanistic link to the observed developmental and functional phenotypes. This aligns with studies showing that Ca^2+^ dependent pathways are relevant to astrocyte growth and structural maturation^67,68^. Appropriate outgrowth and process elaboration is critical for proper synaptic ensheathment and gliotransmitter release, and loss or reduction of these functional processes could underlie deficits in astrocyte-neuron signaling, thus impairing synaptic integrity. Interestingly, while overall volume and surface area were reduced in IP3R2 KOs, the geometric parameters of astrocyte shape such as sphericity and ellipticity were preserved. This suggests that IP3R2-mediated Ca^2+^ signaling selectively regulates growth rather than global cytoskeletal patterning mechanisms. Future work will determine whether these morphological deficits disrupt functional astrocyte–synapse interactions *in vivo* and how they relate to gliotransmission or synapse specificity.

In summary, in the present study we demonstrate that IP3R2 mediated astrocytic Ca^2+^ signaling is an essential intermediary in the development and function of excitatory synapses in the mouse visual system. Given that astrocytic Ca^2+^ signaling is highly heterogeneous spatially, temporally, and mechanistically^15,27,28,30,36,69–72^, our findings provide direct evidence for the contribution of a *defined signaling pathway* (ER store-released Ca^2+^ via IP3R2) within a *specific circuit* (visual circuit), and during a critical *developmental period*. By establishing a role for IP3R2 mediated astrocytic Ca^2+^ signaling in excitatory synapse maturation and visually driven behaviors, this work advances current understanding of how astrocyte-neuron interactions shape circuit development and maturation. These findings underscore the need to better understand how astrocytes integrate diverse Ca^2+^ signals to coordinate their functions across circuits. To fully appreciate and comprehend these complex relationships, studies combining astrocyte specific manipulations, *in vivo* imaging, and computational modeling will be essential to our understanding of how distinct astrocytic Ca^2+^ signals contribute to synapse development, circuit function, and behavior.

## MATERIALS AND METHODS

### Animals

All animal work was approved by the Texas A&M University (TAMU) Institutional Animal Care and use Committee (IACUC) (Animal Use Protocol #2023–0244).

Mice were maintained under standard housing conditions on a 12-hour light:dark cycle with *ad libitum* access to food and water. Both female and male mice from newborn through adult were used in experiments. The following mouse lines were used: Wild-type (WT; C57Bl6/J) were purchased from Jackson Labs and bred in-house (Jax #000664). Mice were used for breeding and backcrossing, and as controls. IP3R2 KO (Itpr2^tm1.1Chen^) was originally obtained from the Ju Chen lab^73^ and maintained on C57BL6/J background as KO X KO or het X het breeding schemes. To generate experimental groups, KO mice were compared with WT collected at the same developmental stages and time-of-day.

### Mouse tissue collection

#### Histology:

Tissue was collected at post-natal days (P) 7, 14, 16, 28–30. Mice were anaesthetized by I.P. injection of 100 mg/kg Ketamine /20 mg/kg Xylazine mix (obtained from Comparative Medicine program at TAMU) and transcardially perfused with PBS, then 4% PFA at room temperature. Brains were removed and incubated in 4% PFA overnight at 4C, then washed 3 × 5 min with PBS, and cryoprotected in 30% sucrose for 2–3 days, then embedded in TFM media (VWR # 100496–345), frozen in dry ice-ethanol slurry solution, and stored at −80C until use. Brains were sectioned using a cryostat (Leica CM1950) in sagittal or coronal orientations depending on experimental needs at a slice thickness of 18–20 μm, or 100 μm. Sections were either mounted on Superfrost plus slides (Fisher #1255015) or kept in PBS (as floating sections) and either used immediately for histological procedures (Immunohistochemistry; IHC) or stored at −80C /4C for later use. 3–5 mice from each sex and age group were used. For each mouse, 2–3 sections were imaged and analyzed.

#### Western Blot /qPCR:

Mice were anaesthetized by I.P. injection of 100 mg/kg Ketamine /20 mg/kg Xylazine mix and then decapitated. Brains were rapidly removed and the VC dissected in ice-cold PBS, flash frozen and kept at −80C until use. 3–5 samples for each condition were analyzed.

### Immunohistochemistry

#### Slide mounted sections:

Sections were blocked for 1 hour at room temperature in blocking buffer containing antibody buffer (100 mM L-lysine and 0.3% Triton X-100 in PBS) supplemented with 10% heat-inactivated normal goat serum. Primary antibodies diluted in antibody buffer with 5% goat serum were incubated shaking overnight at 4°C. The following day, sections were washed 3 × 5 min with PBS with 0.2% Triton X-100 and secondary antibodies conjugated to Alexa Fluor were applied for 2 hours at room temperature. Slides were mounted in SlowFade Gold media with DAPI (LifeTech #S36939), covered with #1.5 glass coverslips (Fisher #12544E), and sealed with clear nail polish.

#### Free-floating sections:

Sections were blocked for 1 hour shaking at room temperature in blocking buffer containing antibody buffer (100 mM L-lysine and 0.3% Triton X-100 in PBS) supplemented with 10% heat-inactivated normal goat serum. Primary antibodies diluted in antibody buffer with 5% goat serum were incubated shaking for 24 hours at 4°C. The following day, sections were washed 3 × 15 min with PBS with 0.2% Triton X-100 and secondary antibodies conjugated to Alexa Fluor were applied for 2 hours shaking at room temperature. Slides were mounted in SlowFade Gold media with DAPI (LifeTech #S36939), covered with #1.5 glass coverslips (Fisher #12544E), and sealed with clear nail polish.

The following primary antibodies were used: Rb anti IP3R2 (Alomone labs #ACC-116, 1:250), Gp anti-VGLUT1 (Millipore #AB5905, 1:1000), Gp anti-VGLUT2 (Millipore #AB2251 1:1000), Rb anti-PSD95 (Thermo-Fisher #516900 1:250), Gp anti-VGAT (Synaptic Systems #131004 1:250), Rb anti-Gephyrin (Synaptic Systems #147008 1:500), Rb anti-NF200 (Millipore Sigma #N4142 1:400), Chk anti-GFP (Invitrogen A10262 1:1000), Rb anti-S100β (Abcam #AB52642, 1:100), Ms anti-NeuN (Millipore #MAB377, 1:100). The following secondary antibodies were used: Gt anti-Rb Alexa-488 (Invitrogen #A11043), Gt anti-Gp Alexa-555 (Invitrogen #A21435), Gt antiChk Alexa-488 (Invitrogen #A32931), Gt anti-Rb Alexa-594 (Invitrogen #A11037), Gt anti-Gp Alexa-594 (Invitrogen #A11076), Gt anti-Ms Alexa-647 (Invitrogen #A21236). All secondary antibodies were used at 1:500 dilution.

### Microscopy and imaging

#### Fluorescent microscopy:

Was performed to image the expression of IP3R2 (Fig. 1, Fig. S1), developmental changes in NF200 (Fig. 2), and c-FOS expression (Fig. 4, Fig. S4) using a Leica THUNDER Imager with LED3 light source, and sCMOS camera (Leica DFC9000) at 20X magnification. Single plane images (2048 × 2048 pixels; 665.49 × 665.49 μm) containing the visual cortex or hippocampus (for NF200) were taken for IP3R2 validation and NF200 studies (Fig.1, Fig. S1, Fig. 3). For c-FOS imaging experiments (Fig. 4, Fig. S4), z stack images (2048 × 2048 pixels; 665.49 × 665.49 × 6 μm) containing the visual cortex, hippocampus, dorsal lateral geniculate nucleus, or superior colliculus were acquired for each brain section separately. A tile image was taken to encompass all visual cortex neuronal layers (2 tiles). Example images are shown as a single z plane image from the same location in the stack for each genotype. Thunder processing (Leica LASX software) was performed using default parameters for single plane imaging (Instant Computational Clearing) to increase resolution and image clarity in the same way for all images.

#### Confocal microscopy:

Was used to image synaptic proteins (Fig. 1, Fig. S1, Fig. S2, Fig. 3) and Lck-eGFP labeled astrocytes (Fig. 6, Fig. S6). Imaging was performed using a Zeiss LSM 900 upright confocal laser scanning microscope with Airyscan2. Synaptic imaging of VGLUT/PSD95 and VGAT/Gephyrin puncta (Fig.1, Fig. S1, Fig. 3) were acquired at 63X magnification. For each section, 1024 × 1024 pixels (101.4 × 101.4 × 2.79 μm) thick z stack image was obtained (pixel size 0.09 × 0.09 × 0.31 μm; 10 slices per 2.79 μm stack). Astrocyte morphological imaging (Fig. 6) was acquired at 63X magnification using the Airyscan2 module, 1834 × 1834 pixels (78.01 × 78.01 XY μm), and 27–47 μm stack to ensure imaging included the entire cell, (pixel size 0.043 × 0.043 × 0.15 μm). Astrocytes located in layer 1 of the VC were selected for imaging if they did not directly connect to the pia (limitans). For validation of Lck-eGFP localization with astrocyte marker S100β (Fig. S6), 20X magnification was used to acquire 512 × 512 pixels (247.35 × 247.35 ×15 μm) z stacks (pixel size 0.48 × 0.48 × 0.31 μm). Example images of synaptic proteins (Fig. 1, Fig. S1, Fig. 3) are shown as a single z plane image from the same location of the stack for each genotype, while example images for Lck-eGFP labeled astrocytes are shown as maximum intensity projections.

### Image analysis and quantification

Image analysis was done with ImageJ (NIH) or Imaris (Bitplane) software as described:

#### Quantification of pre /postsynaptic puncta and synapses:

(Fig. 1, Fig. S1, Fig. S2, Fig. 3) Was performed as previously described^4^ on 3D images using Imaris software (Bitplane). The surfaces function was used to create volumes for VGLUT or VGAT signals, while the spots function was used to render PSD95 or Gephyrin. Total number of surfaces/spots was obtained to obtain pre- and post- synaptic puncta count, and synapses were quantified by counting post-synaptic ‘spots’ located within ≤0.2 μm of a presynaptic surface. Number of total and colocalized puncta were compared between the experimental groups. A minimum of three sections per mouse were imaged and analyzed for each brain region, and the experiment was repeated in at least five WT and KO pairs. Volumes were computed from the surfaces generated for VGLUT1, VGLUT2 or VGAT and exported from Imaris.

#### Quantification of IP3R2 signal:

(Fig. 1, Fig. S1) Images labeled with IP3R2 and S100β (to label astrocytes) or Neun (to label neurons) were analyzed using a semi-automated custom-made macro in ImageJ^4^. For each image, VCs were manually cropped and saved to a new file. For each of the different signals (IP3R2, S100β or Neun), images were manually thresholded in the same way for each section, and the IP3R2 signal area within S100β or Neun ROIs was calculated as thresholded area. Resulting thresholded areas of the IP3R2 signal were normalized to the total area to obtain thresholded area per mm^2^.

#### Quantification of axonal density:

(Fig. 2, Fig. S2) Images labeled with anti-NF200 antibody (to label axons) and DAPI (to label nuclei) were analyzed using a semi-automated custom-made macro in ImageJ. For each image, VCs were manually cropped and saved to a new file. Images were thresholded in the same way for each section for NF200 signal, and the ‘analyze particles’ function was used with a size range of 10–150 pixels to quantify DAPI. Resulting average thresholded area was normalized to the total area analyzed to obtain thresholded area per mm^2^, and cell counts were normalized to the total area to obtain the number of cells per mm^2^.

#### Quantification of c-FOS:

(Fig. 4, Fig. S4) Images labeled with anti-c-FOS antibody and DAPI (to label nuclei) were analyzed using a semi-automated custom-made macro in ImageJ. Maximum intensity projection images were generated from z stacks, cropped by cortical layer (VC) or region (dLGN, SC, CA1, DG) and saved as individual files for analysis. Sections containing dLGN were co-stained for VGLUT2 to identify regional boundaries. Each channel was thresholded using the dark-background auto-threshold method, followed by manual adjustment by the user. For c-FOS, particles within a size range of 25–350 pixels were detected, and the total count was measured within ROIs. For DAPI, particles sized 10–150 pixels were segmented following watershed separation and counted. Cell counts were normalized to the total area to obtain the number of cells per mm^2^

#### Quantification of astrocyte morphology:

(Fig. 6) Was performed on 3D images using Imaris. The surface function was used to create volumes using the absolute intensity filter and cropped to remove partial signal from neighboring astrocytes within the field of view. Remaining surfaces were unified to generate outputs for total volume and area as well as sphericity and ellipticity. A minimum of 5 cells from 3 different sections in at least 5 WT and KO pairs were imaged and analyzed.

#### Quantification of GfaABC1D-Lck-GFP colocalization with S100β:

(Fig. S6) Images containing astrocytes expressing Lck-eGFP were co-labeled with antibody for S100β (to mark astrocytes) and analyzed using a semi-automated custom-made macro in ImageJ. For each of the different signals (Lck-eGFP, S100β), images were thresholded in the same way for each section, and the “cell counter” tool was used for manual count of each of the cell types, as well as the colocalized cells. Resulting cell and colocalization counts were summarized to obtain % overlapping cells.

### Adeno associated viral (aav) vectors and intracerebroventricular (icv) injection

GfaABC1D-Lck-GFP construct (VectorBuilder, #VB240226–1200bvh) was purchased from VectorBuilder as viral particles (AAV5), aliquoted and stored at −80C until use.

ICV injection was performed as previously described^74–76^. P1 mouse pups were removed from their home cage and placed in a warmed holding cage. Pups were individually cryo-anaesthetized in ice for 5 minutes (placed inside a glove) before being transferred to a surgical pad over a back-lit surface. The ventricles were identified by measuring the distance 2/5^ths^ from the lambda suture to the eye. 1–2 μL of AAV5-GfaABC1D-Lck-GFP (VectorBuilder, #VB240226–1200bvh) diluted to 2 × 10^9^ gc/mL in saline was drawn into the barrel of a 10 μl glass syringe (Hamilton #7653–01) fitted with a 32G injection needle (Hamilton #7803–04). With the pup’s head upright and secured by hand, the injection site was swabbed with 70% ethanol, and the tip of the needle was positioned perpendicular to the injection site and inserted ~3 mm before the plunger was manually depressed. The syringe was held in place for 10 seconds to avoid ejection, and then carefully withdrawn. This procedure was performed for both ventricles. After injection, pups were returned to the warmed holding cage for immediate recovery, then to their home cage for two weeks before brain tissue collection.

### Light pulse assay

P16 mice (aged to ensure eyes were open for at least 24 hours before light pulse experiments) were habituated in cages placed within darkened boxes (32W × 38L × 32 × 54.6H cm) for 30 minutes prior to experimentation, then mice were moved (individually) to either a control cage within a darkened box, or an experimental cage in a separate box fitted with 3 white LED light strips (110 lumen each). Experimental mice were subjected to 330 lumen (~2713 lux) light for 20 minutes, followed by immediate anesthesia by Ketamine/Xylazine injection and perfusion as described (see Mouse tissue section). Control mice remained in dark condition for 20 minutes before tissue collection. The light pulse procedure was performed for all mice between ZT16 – ZT17 after four hours of sustained darkness (ZT12-ZT16) under standard housing conditions.

### Visually evoked defensive behavioral assay

The visually triggered looming shadow test was performed according to published protocols with modifications^47,49^. The setup consists of a rectangular arena (17W × 40L × 20H cm) with a plastic hut (11 cm in diameter, 5 cm height) placed in one corner of the cage serving as shelter for mice to escape to. The hut used is identical to that used in the mouse home cages to facilitate quicker familiarization and habituation to the testing environment. A computer monitor is mounted 40 cm above the arena floor, connected to a computer through which the looming stimulus is delivered. A video camera is placed above the cage to record mouse movement during the stimulus for offline analysis. The test was administered over 3 consecutive days and consisted of a habituation day (day 0), followed by 2 consecutive testing days (see Fig. 5A). During habituation, mice are placed in the testing arena one at a time, and allowed to explore for 13 minutes, without the looming stimulus played. On each testing day, mice are placed in the arena and subjected to 5 trials of the looming stimulus. The first stimulus is played after a minimum of 3 minutes of free exploration, with a minimum of 1 minute interval between subsequent stimuli, during which time mice resume exploratory behavior. Mice spend a total of 13 minutes in the arena on testing days, then are returned to home cages even if they did not receive 5 trials. Stimulus is played only if a mouse is outside the shelter and engaged in exploratory behavior. Day 2 is identical to Day 1, occurring ~24 hours later. Mice are returned to the vivarium between testing days.

#### The looming stimulus:

A 9 second video showing an expanding black disc (2–20cm in diameter) on a white background is played to mimic a looming threat (file available from^49^; see Fig. 5B). The stimulus begins when a black disc (2 cm) appears on the computer screen (~3.5 seconds), rapidly expands to 20 cm diameter (~2 seconds) and remains fully expanded for ~3.5 seconds. This stimulus has been thoroughly characterized in previous studies to elicit robust defensive responses in mice^47,49^.

#### Quantification of responses and kinematics:

1. Defensive behavioral scoring – was done by an experimenter either live during the testing, or offline from recorded videos. For each stimulus, 4 types of responses are recorded: “Freeze” – a mouse’s snout and body are immobile for a minimum of 1 second; “Escape” – the mouse is moving towards and entering the shelter; “Freeze+Escape” – a period of immobility followed by movement and entering the shelter; “No response” – mouse continues exploratory behavior or movement with no directed change due to stimulus.
2. Kinematics analysis – was done by using DeepLabCut^77,78^ trained custom pose estimation model for tracking mice during behavioral experiments, which was applied to the video recordings to generate X-Y coordinates for each frame. The resulting data was used as input by BehaviorDEPOT^79^ to calculate the velocities (computed using the Pythagorean Theorem: √[(dx/dt)^2^ + (dy/dt)^2^]). Custom MATLAB and Python scripts were used to extract kinematic data from the entire recording and from specific frame ranges corresponding to the presentation of the looming stimulus, respectively. The scripts (GitHub_scripts) and the DeepLabCut model (DLC model) are available in the appropriate links.

#### Exclusion criteria:

Mice that missed more than 2 stimuli (e.g. remaining inside the shelter/failure to explore) on either testing day were removed from the study.

### Western blot

Samples were heated in reducing sample buffer (Thermo #39000) for 45 min at 55°C. For tissue lysates, 5 μg/lane was loaded. Samples were resolved on Bolt 4–12% Bis-Tris gels (Invitrogen #NW04125BOX) for 1 hour and 15 min at 150 V. Proteins were transferred to Immobilon-FL PVDF membranes (Millipore #IPFL00010) at 20 V for 1 hr, then blocked in 1% casein (Bio-Rad #1610782) in TBS (Bioworld #10530027–2) blocking buffer for 1 hr at room temperature on a shaker. Primary antibodies were applied overnight at 4C diluted in blocking buffer. The antibodies used were Rb anti-IP3R2 (Alomone labs # ACC-116, 1:500), and Ms anti-GAPDH (Cell Signaling Technology #97166T, 1:2000). The next day, membranes were washed 3 × 10 min with TBS-0.1% Tween (Promega #PRH5152) and secondary antibodies Gt anti-Rb Alexa-680 (Invitrogen #A21109) and Gt anti-Ms-Alexa-800 (Invitrogen #PIA32730) were applied for 2 hr at room temperature (dilution 1:10,000). Bands were visualized using the LI-COR Odyssey CLx Infrared Imager and band intensity analyzed using the LI-COR Image Studio software.

### Quantitative real-time PCR (qPCR)

Total RNA was isolated from 15–20 mg of flash-frozen cortical tissue using RNeasy Plus Mini Kit (Qiagen #74136). RNA yield and purity was analyzed by NanoDrop ND-1000 spectrophotometer (Nanodrop technologies, USA), and first-strand cDNA was synthesized from 1μg total RNA using SuperScript VILO MasterMix (Invitrogen #11755–050) following the manufacturer’s instructions. Quantitative real time PCR was executed using a Bio-Rad CFX Opus 96 Real-Time PCR System (Bio-Rad, USA). For each reaction the 20 μL reaction mixture contained 1 μL cDNA template, 8 μL molecular biology grade water, 1 μL primer mix (forward and reverse, 10 μM), and 10 μL 2X SYBR Green PCR Master Mix (Applied Biosystems #4364344). RNA levels were quantified for relative expression using the ΔCT method with *Gapdh* as a reference transcript. PCR reactions were performed in triplicate for each cDNA sample. Primers were designed from nucleotide sequences obtained using NCBI BLAST (http://blast.ncbi.nlm.nih.gov/Blast.cgi) and synthesized by Integrated DNA Technologies. Primer pairs used are as follows: GAPDH-F CATCACTGCCACCCAGAAGACTG, GAPDH-R ATGCCAGTGAGCTTCCCGTTCAG; IP3R2-F AGACTGCCTGTTCAAGGTGTG, IP3R2-R ATGGTTCCCCTGTTTCGCCTG; CST3-F CATACAGGTGGTCAGAGCTCGT, CST3-R CTGGTCATGGAAAGGACAGTCAG; FSTL1-F CCACCTTCGCCTCTAACTCG, FSTL1-R ATCGTTTCCACATCGTGCTC; GLUD1-F TCCGTTACAGCACTGACGTGAG, GLUD1-R ACGCCTGCTTTAGCACTGCCAA; GRIN2B-F CTGGTGACCAATGGCAAGCATG, GRIN2B-R GGCACAGAGAAGTCAACCACCT; KCNK2-F GCTATACTGCAGGAGTGGCG, KCNK2-R AGGAGAATGAGAGCCTCGGT; LGR4-F GTGCATTTTGGGGGTGTGAC, LGR4-R TGCTGCATCTGTAGCACCTTT; MAPK10-F AGCTGGATGAAAGGGAGCAC, MAPK10-R ACTGCTGCACCTGAAGGC; OGT-F GGCTATGTGAGTTCTGACTTCGG, OGT-R GATTGGCTTCCGCCATCACCTT.

### Statistical analysis

Data is shown as mean ± s.e.m, median with range, or percentages, as indicated in each Figure legend. Descriptive statistics are reported in the text as 2 or more numbers after the decimal point unrounded. Statistical analysis was performed using Prism software (Graphpad). Multiple group comparisons were done using one-way analysis of variance (ANOVA) with post hoc Tukey’s, Šidák or Dunn’s tests for normally distributed data, or Kruskal-Wallis ANOVA on ranks with post hoc Dunn’s test for data that did not exhibit normal distribution. Pairwise comparisons were done by t-test (normal distribution) or Mann-Whitney rank sum test (non-normal distribution). Each dataset was tested for normality (with Shapiro-Wilks test) to ensure the correct statistical test is used. Fisher’s exact test was used to compare percentages of responding mice (Fig. 5, Fig. S5). Two sample Kolmogorov-Smirnov test was used to assess differences between cumulative frequency distributions. Analysis was completed blind to genotype when possible. Sample sizes, statistical tests used, and significance values are presented in each Figure and Figure legend. P Value ≤ 0.05 was considered statistically significant. All the data including the input values (descriptive statistics) and full statistical analysis information (including all pairwise comparisons, ANOVA F values etc.) are detailed in the supplemental Table S1 for each Figure as labeled.

## Supplementary Material

Document S1. 5 Supplemental figures. Figures S1, S2, S4, S5, S6.

Supplemental table Table S1.

This is a list of supplementary files associated with this preprint. Click to download.

- ImrieTableS1.xlsx
- ImrieetalSupplementalDocumentS12025.pdf

## Data Availability

Requests for further information and resources should be directed to and will be fulfilled by the lead contact, Isabella Farhy-Tselnicker (ifarhy@bio.tamu.edu).

## References

[1] Chalmers N., Masouti E. & Beckervordersandforth R. Astrocytes in the adult dentate gyrus-balance between adult and developmental tasks. Mol Psychiatry 29, 982–991 (2024). 10.1038/s41380-023-02386-4 38177351 PMC11176073

[2] Farhy-Tselnicker I. & Allen N. J. Astrocytes, neurons, synapses: a tripartite view on cortical circuit development. Neural Development 13, 7 (2018). 10.1186/s13064-018-0104-y 29712572 PMC5928581

[3] Tan C. X., Burrus Lane C. J. & Eroglu C. Role of astrocytes in synapse formation and maturation. Curr Top Dev Biol 142, 371–407 (2021). 10.1016/bs.ctdb.2020.12.010 33706922

[4] Farhy-Tselnicker I. Activity-dependent modulation of synapse-regulating genes in astrocytes. Elife 10 (2021). 10.7554/eLife.70514 PMC8497060 34494546

[5] Xie Y. Astrocyte-neuron crosstalk through Hedgehog signaling mediates cortical synapse development. Cell Rep 38, 110416 (2022). 10.1016/j.celrep.2022.110416 35196485 PMC8962654

[6] Duman R. S., Sanacora G. & Krystal J. H. Altered Connectivity in Depression: GABA and Glutamate Neurotransmitter Deficits and Reversal by Novel Treatments. Neuron 102, 75–90 (2019). 10.1016/j.neuron.2019.03.013 30946828 PMC6450409

[7] Guang S. Synaptopathology Involved in Autism Spectrum Disorder. Front Cell Neurosci 12, 470 (2018). 10.3389/fncel.2018.00470 30627085 PMC6309163

[8] Bernard C. in Jasper’s Basic Mechanisms of the Epilepsies (eds Noebels J. L. ) (2012). 22787592

[9] Li H. Laminar and columnar development of barrel cortex relies on thalamocortical neurotransmission. Neuron 79, 970–986 (2013). 10.1016/j.neuron.2013.06.043 24012009 PMC3768017

[10] Blue M. E. & Parnavelas J. G. The formation and maturation of synapses in the visual cortex of the rat. II. Quantitative analysis. J Neurocytol 12, 697–712 (1983). 10.1007/BF01181531 6619907

[11] Boisvert M. M., Erikson G. A., Shokhirev M. N. & Allen N. J. The Aging Astrocyte Transcriptome from Multiple Regions of the Mouse Brain. Cell Rep 22, 269–285 (2018). 10.1016/j.celrep.2017.12.039 29298427 PMC5783200

[12] Imrie G., Gray M. B., Raghuraman V. & Farhy-Tselnicker I. Gene Expression at the Tripartite Synapse: Bridging the Gap Between Neurons and Astrocytes. Adv Neurobiol 39, 95–136 (2024). 10.1007/978-3-031-64839-7_5 39190073 PMC12289143

[13] Faust T. E. Glial Control of Cortical Neuronal Circuit Maturation and Plasticity. J Neurosci 44 (2024). 10.1523/JNEUROSCI.1208-24.2024 PMC11450532 39358028

[14] Adamsky A. Astrocytic Activation Generates De Novo Neuronal Potentiation and Memory Enhancement. Cell 174, 59–71 e14 (2018). 10.1016/j.cell.2018.05.002 29804835

[15] Ahrens M. B., Khakh B. S. & Poskanzer K. E. Astrocyte Calcium Signaling. Cold Spring Harb Perspect Biol 16 (2024). 10.1101/cshperspect.a041353 PMC11444304 38768971

[16] Bai Y. Revisiting astrocytic calcium signaling in the brain. Fundam Res 4, 1365–1374 (2024). 10.1016/j.fmre.2023.11.021 39734522 PMC11670731

[17] Imrie G. & Farhy-Tselnicker I. Astrocyte regulation of behavioral outputs: the versatile roles of calcium. Front Cell Neurosci 19, 1606265 (2025). 10.3389/fncel.2025.1606265 40443710 PMC12119555

[18] Cho W. H. Hippocampal astrocytes modulate anxiety-like behavior. Nat Commun 13, 6536 (2022). 10.1038/s41467-022-34201-z 36344520 PMC9640657

[19] Peyton L. In vivo calcium extrusion from accumbal astrocytes reduces anxiety-like behaviors but increases compulsive-like responses and compulsive ethanol drinking in mice. Neuropharmacology 268, 110320 (2025). 10.1016/j.neuropharm.2025.110320 39842625 PMC11830519

[20] Rupprecht P. Centripetal integration of past events in hippocampal astrocytes regulated by locus coeruleus. Nat Neurosci 27, 927–939 (2024). 10.1038/s41593-024-01612-8 38570661 PMC11089000

[21] Suthard R. L. Chronic Gq activation of ventral hippocampal neurons and astrocytes differentially affects memory and behavior. Neurobiol Aging 125, 9–31 (2023). 10.1016/j.neurobiolaging.2023.01.007 36801699

[22] Wang Q. Impaired calcium signaling in astrocytes modulates autism spectrum disorder-like behaviors in mice. Nat Commun 12, 3321 (2021). 10.1038/s41467-021-23843-0 34059669 PMC8166865

[23] Yu X. Reducing Astrocyte Calcium Signaling In Vivo Alters Striatal Microcircuits and Causes Repetitive Behavior. Neuron 99, 1170–1187 e1179 (2018). 10.1016/j.neuron.2018.08.015 30174118 PMC6450394

[24] Bazargani N. & Attwell D. Astrocyte calcium signaling: the third wave. Nat Neurosci 19, 182–189 (2016). 10.1038/nn.4201 26814587

[25] Sherwood M. W., Arizono M., Panatier A., Mikoshiba K. & Oliet S. H. R. Astrocytic IP(3)Rs: Beyond IP(3)R2. Front Cell Neurosci 15, 695817 (2021). 10.3389/fncel.2021.695817 34393726 PMC8363081

[26] Srinivasan R. Ca(2+) signaling in astrocytes from Ip3r2(−/−) mice in brain slices and during startle responses in vivo. Nat Neurosci 18, 708–717 (2015). 10.1038/nn.4001 25894291 PMC4429056

[27] Agarwal A. Transient Opening of the Mitochondrial Permeability Transition Pore Induces Microdomain Calcium Transients in Astrocyte Processes. Neuron 93, 587–605 e587 (2017). 10.1016/j.neuron.2016.12.034 28132831 PMC5308886

[28] Ahmadpour N., Kantroo M. & Stobart J. L. Extracellular Calcium Influx Pathways in Astrocyte Calcium Microdomain Physiology. Biomolecules 11 (2021). 10.3390/biom11101467 PMC8533159 34680100

[29] Denizot A., Arizono M., Nagerl U. V., Berry H. & De Schutter E. Control of Ca(2+) signals by astrocyte nanoscale morphology at tripartite synapses. Glia 70, 2378–2391 (2022). 10.1002/glia.24258 36097958 PMC9825906

[30] Lia A. Calcium Signals in Astrocyte Microdomains, a Decade of Great Advances. Front Cell Neurosci 15, 673433 (2021). 10.3389/fncel.2021.673433 34163329 PMC8216559

[31] Shigetomi E., Jackson-Weaver O., Huckstepp R. T., O’Dell T. J. & Khakh B. S. TRPA1 channels are regulators of astrocyte basal calcium levels and long-term potentiation via constitutive D-serine release. J Neurosci 33, 10143–10153 (2013). 10.1523/JNEUROSCI.5779-12.2013 23761909 PMC3682388

[32] Guillot de Suduiraut I., Grosse J., Ramos-Fernandez E., Sandi C. & Hollis F. Astrocytic release of ATP through type 2 inositol 1,4,5-trisphosphate receptor calcium signaling and social dominance behavior in mice. Eur J Neurosci 53, 29732985 (2021). 10.1111/ejn.14892 32609904

[33] Jego P., Pacheco-Torres J., Araque A. & Canals S. Functional MRI in mice lacking IP3-dependent calcium signaling in astrocytes. J Cereb Blood Flow Metab 34, 1599–1603 (2014). 10.1038/jcbfm.2014.144 25099754 PMC4269735

[34] Petravicz J., Boyt K. M. & McCarthy K. D. Astrocyte IP3R2-dependent Ca(2+) signaling is not a major modulator of neuronal pathways governing behavior. Front Behav Neurosci 8, 384 (2014). 10.3389/fnbeh.2014.00384 25429263 PMC4228853

[35] Petravicz J., Fiacco T. A. & McCarthy K. D. Loss of IP3 receptor-dependent Ca2+ increases in hippocampal astrocytes does not affect baseline CA1 pyramidal neuron synaptic activity. J Neurosci 28, 4967–4973 (2008). 10.1523/JNEUROSCI.5572-07.2008 18463250 PMC2709811

[36] Khakh B. S. & McCarthy K. D. Astrocyte calcium signaling: from observations to functions and the challenges therein. Cold Spring Harb Perspect Biol 7, a020404 (2015). 10.1101/cshperspect.a020404 25605709 PMC4382738

[37] Veiga A. Calcium-Dependent Signaling in Astrocytes: Downstream Mechanisms and Implications for Cognition. J Neurochem 169, e70019 (2025). 10.1111/jnc.70019 39992167

[38] Lines J., Martin E. D., Kofuji P., Aguilar J. & Araque A. Astrocytes modulate sensory-evoked neuronal network activity. Nat Commun 11, 3689 (2020). 10.1038/s41467-020-17536-3 32704144 PMC7378834

[39] Zhi J. J. Insufficient Oligodendrocyte Turnover in Optic Nerve Contributes to Age-Related Axon Loss and Visual Deficits. J Neurosci 43, 1859–1870 (2023). 10.1523/JNEUROSCI.2130-22.2023 36725322 PMC10027114

[40] Benn S. C., Costigan M., Tate S., Fitzgerald M. & Woolf C. J. Developmental expression of the TTX-resistant voltage-gated sodium channels Nav1.8 (SNS) and Nav1.9 (SNS2) in primary sensory neurons. J Neurosci 21, 6077–6085 (2001). 10.1523/JNEUROSCI.21-16-06077.2001 11487631 PMC6763192

[41] Sawant L. A., Hasgekar N. N. & Vyasarayani L. S. Developmental expression of neurofilament and glial filament proteins in rat cerebellum. Int J Dev Biol 38, 429–437 (1994). 7848826

[42] Wei J. R. Identification of visual cortex cell types and species differences using single-cell RNA sequencing. Nat Commun 13, 6902 (2022). 10.1038/s41467-022-34590-1 36371428 PMC9653448

[43] Ellis E. M., Gauvain G., Sivyer B. & Murphy G. J. Shared and distinct retinal input to the mouse superior colliculus and dorsal lateral geniculate nucleus. J Neurophysiol 116, 602–610 (2016). 10.1152/jn.00227.2016 27169509 PMC4982907

[44] Schiapparelli L. M. The Retinal Ganglion Cell Transportome Identifies Proteins Transported to Axons and Presynaptic Compartments in the Visual System In Vivo. Cell Rep 28, 1935–1947 e1935 (2019). 10.1016/j.celrep.2019.07.037 31412257 PMC6707540

[45] Ahmadlou M., Zweifel L. S. & Heimel J. A. Functional modulation of primary visual cortex by the superior colliculus in the mouse. Nat Commun 9, 3895 (2018). 10.1038/s41467-018-06389-6 30254324 PMC6156231

[46] White B. J., Kan J. Y., Levy R., Itti L. & Munoz D. P. Superior colliculus encodes visual saliency before the primary visual cortex. Proc Natl Acad Sci U S A 114, 9451–9456 (2017). 10.1073/pnas.1701003114 28808026 PMC5584409

[47] Daviu N. Paraventricular nucleus CRH neurons encode stress controllability and regulate defensive behavior selection. Nat Neurosci 23, 398–410 (2020). 10.1038/s41593-020-0591-0 32066984

[48] Narushima M., Agetsuma M. & Nabekura J. Development and experience-dependent modulation of the defensive behaviors of mice to visual threats. J Physiol Sci 72, 5 (2022). 10.1186/s12576-022-00831-7 35255805 PMC10717832

[49] Daviu N. Visual-looming Shadow Task with in-vivo Calcium Activity Monitoring to Assess Defensive Behaviors in Mice. Bio Protoc 10, e3826 (2020). 10.21769/BioProtoc.3826 PMC7842776 33659478

[50] Shamash P. & Branco T. Protocol to Study Spatial Subgoal Learning Using Escape Behavior in Mice. Bio Protoc 12, e4443 (2022). 10.21769/BioProtoc.4443 PMC9257842 35864903

[51] Vale R., Evans D. A. & Branco T. Rapid Spatial Learning Controls Instinctive Defensive Behavior in Mice. Curr Biol 27, 1342–1349 (2017). 10.1016/j.cub.2017.03.031 28416117 PMC5434248

[52] Cheng Y. T. Inhibitory input directs astrocyte morphogenesis through glial GABA(B)R. Nature 617, 369–376 (2023). 10.1038/s41586-023-06010-x 37100909 PMC10733939

[53] Wu L. The cell-surface shared proteome of astrocytes and neurons and the molecular foundations of their multicellular interactions. Neuron (2025). 10.1016/j.neuron.2025.05.019 PMC12354340 40499536

[54] Hu Y. T., Tan Z. L., Hirjak D. & Northoff G. Brain-wide changes in excitation-inhibition balance of major depressive disorder: a systematic review of topographic patterns of GABA- and glutamatergic alterations. Mol Psychiatry 28, 3257–3266 (2023). 10.1038/s41380-023-02193-x 37495889

[55] Li G. Revealing excitation-inhibition imbalance in Alzheimer’s disease using multiscale neural model inversion of resting-state functional MRI. Commun Med (Lond) 5, 17 (2025). 10.1038/s43856-025-00736-7 39814858 PMC11735810

[56] Sylvester A. L. Neural excitation/inhibition imbalance and neurodevelopmental pathology in human copy number variant syndromes: a systematic review. J Neurodev Disord 17, 31 (2025). 10.1186/s11689-025-09614-8 40490701 PMC12147258

[57] Liu Y. A Selective Review of the Excitatory-Inhibitory Imbalance in Schizophrenia: Underlying Biology, Genetics, Microcircuits, and Symptoms. Front Cell Dev Biol 9, 664535 (2021). 10.3389/fcell.2021.664535 34746116 PMC8567014

[58] Liu J. Astrocyte dysfunction drives abnormal resting-state functional connectivity in depression. Sci Adv 8, eabo2098 (2022). 10.1126/sciadv.abo2098 36383661 PMC9668300

[59] Cahill M. K. Network-level encoding of local neurotransmitters in cortical astrocytes. Nature 629, 146–153 (2024). 10.1038/s41586-024-07311-5 38632406 PMC11062919

[60] Reitman M. E. Norepinephrine links astrocytic activity to regulation of cortical state. Nat Neurosci 26, 579–593 (2023). 10.1038/s41593-023-01284-w 36997759 PMC10089924

[61] De Franceschi G., Vivattanasarn T., Saleem A. B. & Solomon S. G. Vision Guides Selection of Freeze or Flight Defense Strategies in Mice. Curr Biol 26, 2150–2154 (2016). 10.1016/j.cub.2016.06.006 27498569

[62] Tseng Y. T., Schaefke B., Wei P. & Wang L. Defensive responses: behaviour, the brain and the body. Nat Rev Neurosci 24, 655–671 (2023). 10.1038/s41583-023-00736-3 37730910

[63] Oliveira J. F. & Araque A. Astrocyte regulation of neural circuit activity and network states. Glia 70, 1455–1466 (2022). 10.1002/glia.24178 35460131 PMC9232995

[64] Bojarskaite L. Astrocytic Ca(2+) signaling is reduced during sleep and is involved in the regulation of slow wave sleep. Nat Commun 11, 3240 (2020). 10.1038/s41467-020-17062-2 32632168 PMC7338360

[65] Peng W. Adenosine-independent regulation of the sleep-wake cycle by astrocyte activity. Cell Discov 9, 16 (2023). 10.1038/s41421-022-00498-9 36746933 PMC9902472

[66] Wang F. Distinct astrocytic modulatory roles in sensory transmission during sleep, wakefulness, and arousal states in freely moving mice. Nat Commun 14, 2186 (2023). 10.1038/s41467-023-37974-z 37069258 PMC10110578

[67] Bernardinelli Y. Activity-dependent structural plasticity of perisynaptic astrocytic domains promotes excitatory synapse stability. Curr Biol 24, 1679–1688 (2014). 10.1016/j.cub.2014.06.025 25042585

[68] Chen J. Astrocyte growth is driven by the Tre1/S1pr1 phospholipid-binding G protein-coupled receptor. Neuron 112, 93–112 e110 (2024). 10.1016/j.neuron.2023.11.008 38096817 PMC11073822

[69] Oberheim N. A., Goldman S. A. & Nedergaard M. Heterogeneity of astrocytic form and function. Methods Mol Biol 814, 23–45 (2012). 10.1007/978-1-61779-452-0_3 22144298 PMC3506190

[70] Bindocci E. Three-dimensional Ca(2+) imaging advances understanding of astrocyte biology. Science 356 (2017). 10.1126/science.aai8185 28522470

[71] Bosson A. TRPA1 channels promote astrocytic Ca(2+) hyperactivity and synaptic dysfunction mediated by oligomeric forms of amyloid-beta peptide. Mol Neurodegener 12, 53 (2017). 10.1186/s13024-017-0194-8 28683776 PMC5501536

[72] Benoit L. Astrocytes functionally integrate multiple synapses via specialized leaflet domains. Cell (2025). 10.1016/j.cell.2025.08.036 40997814

[73] Li X., Zima A. V., Sheikh F., Blatter L. A. & Chen J. Endothelin-1-induced arrhythmogenic Ca2+ signaling is abolished in atrial myocytes of inositol-1,4,5-trisphosphate(IP3)-receptor type 2-deficient mice. Circ Res 96, 1274–1281 (2005). 10.1161/01.RES.0000172556.05576.4c 15933266

[74] Glascock J. J. Delivery of therapeutic agents through intracerebroventricular (ICV) and intravenous (IV) injection in mice. J Vis Exp (2011). 10.3791/2968 PMC3227174 21988897

[75] Kawasaki H. Intracerebroventricular and Intravascular Injection of Viral Particles and Fluorescent Microbeads into the Neonatal Brain. J Vis Exp (2016). 10.3791/54164 PMC5091671 27501398

[76] Kim J. Y., Grunke S. D., Levites Y., Golde T. E. & Jankowsky J. L. Intracerebroventricular viral injection of the neonatal mouse brain for persistent and widespread neuronal transduction. J Vis Exp, 51863 (2014). 10.3791/51863 25286085 PMC4199253

[77] Mathis A. DeepLabCut: markerless pose estimation of user-defined body parts with deep learning. Nat Neurosci 21, 1281–1289 (2018). 10.1038/s41593-018-0209-y 30127430

[78] Nath T. Using DeepLabCut for 3D markerless pose estimation across species and behaviors. Nat Protoc 14, 2152–2176 (2019). 10.1038/s41596-019-0176-0 31227823

[79] Gabriel C. J. BehaviorDEPOT is a simple, flexible tool for automated behavioral detection based on markerless pose tracking. Elife 11 (2022). 10.7554/eLife.74314 PMC9398447 35997072

